# Magnetic Nature of Light Transmission through a 5-nm Gap

**DOI:** 10.1038/s41598-018-21037-1

**Published:** 2018-02-09

**Authors:** Hyosim Yang, Dai-Sik Kim, Richard H. Joon-Yeon Kim, Jae Sung Ahn, Taehee Kang, Jeeyoon Jeong, Dukhyung Lee

**Affiliations:** 1 Department of Physics and Astronomy and Center for Atom Scale Electromagnetism, Seoul National University, Seoul, 08826 Republic of Korea; 2 Ames Laboratory, U.S. Department of Energy and Department of Physics and Astronomy, Iowa State University, Ames, Iowa 50011 USA; 3 Bio-medical Photonics Research Center, Korea Photonics Technology Institute, 9 Cheomdan venture-ro 108beon-gil, Gwangju, 61007 Republic of Korea

## Abstract

Slot antennas have been exploited as important building blocks of optical magnetism because their radiations are invoked by the magnetic fields along the axes, as vectorial Babinet principle predicts. However, optical magnetism of a few-nanometer-width slit, for which fascinating applications are found due to the colossal field enhancement but Babinet principle fails due to the nonnegligible thickness, has not been investigated. In this paper, we demonstrated that the magnetic field plays a dominant role in light transmission through a 5-nm slit on a 150-nm-thick gold film. The 5-nm slit was fabricated by atomic layer lithography, and the transmission was investigated for various incident angles by experiment and simulation at 785-nm wavelength. We found that, due to the deep subwavelength gap width, the transmission has the same incident angle dependence as the tangential magnetic field on the metal surface and this magnetic nature of a nanogap holds up to ~100-nm width. Our analysis establishes conditions for nanogap optical magnetism and suggests new possibilities in realizing magnetic-field-driven optical nonlinearities.

## Introduction

An interesting feature of plasmonics is that magnetic field interactions with nanostructures are distinctive from the electric counterparts^1–3^. The magnetic interactions of light, or optical magnetism, enables lots of fascinating phenomena, such as negative refraction^4^ and optical cloaking^5^. Many intuitive understandings of electromagnetic interactions can be given by vectorial Babinet’s principle^6^, which describes the identical actions of a magnetic (electric) field for a planar structure and an electric (magnetic) field for the complementary planar structure.

The most prominent examples of the principle are slot antennas^7,8^. Slot antennas, which are complementary to rod antenna radiating as electric dipoles, act as magnetic dipoles and become an important building block in optical magnetism. With the state-of-the-art fabrication technique, a slot antenna can be fabricated to have a width of ~1 nm and a thickness of ~100 nm^9–12^. Due to the colossal field enhancement in the gap, applications of the few-nanometer-gap can be found in various areas such as ultrasensitive molecular sensing^13^, surface enhanced Raman scattering (SERS)^14^, and nonlinear spectroscopy^15,16^. However, the high aspect ratio (thickness/width) reaching up to several hundreds is incompatible with Babinet’s principle that assumes an infinitesimal screen thickness. Although the applicability of the principle has been confirmed in several slot structures on real metal films of finite permittivities and thicknesses^17–19^, optical magnetism of such a high aspect ratio slot antenna, that is, the determinative role of the magnetic field as in a magnetic dipole, has not been demonstrated.

In this work, we studied the light transmission through a single slit of 5-nm width and 150-nm thickness and found that the transmission through a high aspect ratio nanogap is dominated by the magnetic field, as slot antennas of moderate aspect ratios^2,7,8^. A slit structure can be regarded as a slot antenna of an extreme length, having a simple 2-dimensional geometry enabling easier analysis. As discussed later, the mechanism of the magnetic field dominance is related to the gap plasmon excitation which depends on the gap width. In order to investigate the dominance of the magnetic field, we measured the transmission through the 5-nm slit with various incident angles. Further, we studied the transmission characteristics for different widths by performing finite element method (FEM) simulation to estimate the range where optical magnetism can be realized.

## Results and Discussion

**Figure 1:**
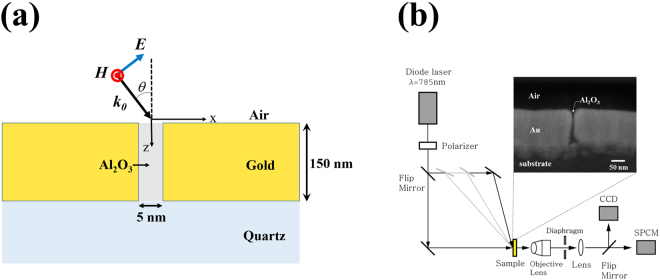
(**a**) Schematic of the problem with a p-polarized oblique incidence. (**b**) Schematic of the experimental setup. The incident angle was adjusted by moving the location of the incidence mirror. The transmitted light through the sample was collected by an objective lens with a NA of 0.25. Lightpath to SPCM or CCD was selected by using a flip mirror. Inset: cross-sectional SEM image of a 5-nm gap.

Figure 1(a) describes the geometry of the sample and the incident light. We investigated the transmission of p-polarized light of which magnetic field is parallel to the gap. Figure 1(b) presents a schematic of our experimental setup. By changing the incidence mirror location, a 785-nm laser beam was obliquely incident on the sample with various incident angles. The transmitted light through the sample was collected in the normal direction by an objective lens whose numerical aperture (NA) is 0.25. A single photon counting module (SPCM) was used for the signal detection. Identifying the gap locations by using a CCD camera, we averaged the signals from three different locations at least. We normalized the oblique incident transmission intensities by the normal incident transmission intensity.

We fabricated a uniform 5-nm slit on a 150-nm-thick gold film using atomic layer lithography previously reported by Chen and Park *et al*.^10^. Details of the nanogap fabrication are covered in the Methods section. For comparison with the 5-nm slit, a 4.6-μm slit was perforated on a gold film with the same thickness by focused ion beam milling.

**Figure 2:**
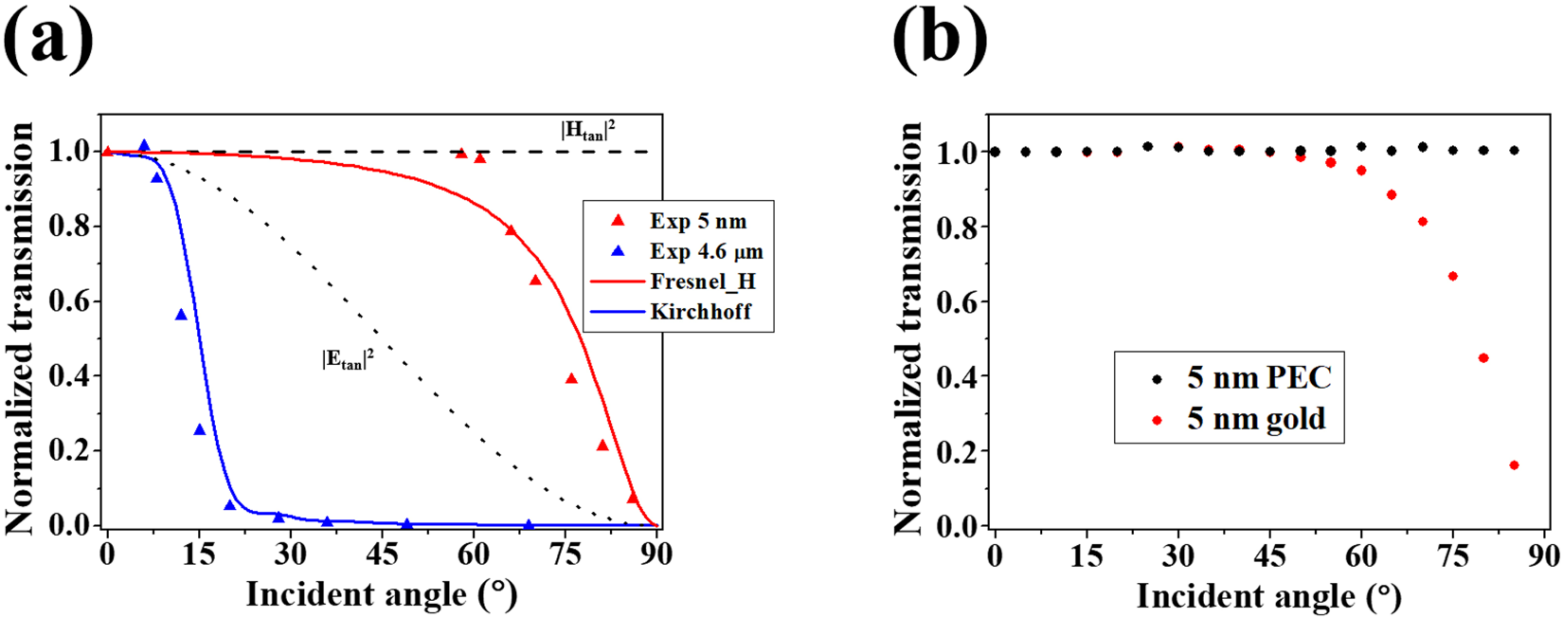
(**a**) Experimental results of transmission intensity versus the incident angle for the 5-nm slit (red triangles) and the 4.6-μm slit (blue triangles). Transmission through the 5-nm slit is almost constant up to 60 degrees and shows similar incident angle dependence with the magnetic field intensity on the metal film given by the Fresnel equations (red solid line). The black dashed and dotted lines indicate the incident tangential magnetic and electric field intensities, respectively. The 4.6-μm slit is in accordance with the Kirchhoff approximation (blue solid line). (**b**) Transmission through the PEC (black circles) and gold (red circles) 5-nm gaps with varying incident angle obtained by FEM simulations.

Dependence of the 5-nm-gap transmission on the incident magnetic and electric fields can be examined from the dependence on the incident angle. Because the incident light is p-polarized, the vertical and tangential components of the incident electric field vary as sine and cosine functions of the incident angle. Meanwhile, the incident magnetic field has only the tangential component that is constant over all incident angles (Fig. 1(a)). According to Babinet’s principle, a slit on a thin PEC film acts as a 2-dimensional magnetic dipole directed along the slit and radiates in proportion to the incident tangential magnetic field. If also the radiation of the 5-nm slit is determined by the incident tangential magnetic field, the transmission would be constant over all incident angles (Fig. 2(a), black dashed line). On the contrary, if the incident tangential electric field determines the gap transmission, the transmission intensity would depend on the incident angle as a cosine squared function (Fig. 2(a), black dotted line). In this point of view, the 5-nm-gap transmission (Fig. 2(a), red triangles) indicates that the magnetic field dominance stands up to around 60 degrees. In contrast, such magnetic field dominance cannot be found for the 4.6-μm slit. Instead, the transmission through the 4.6-μm slit shows good agreement with the Kirchhoff scalar diffraction theory combined with the Kirchhoff approximation (Fig. 2(a), blue triangles and solid line) in which the magnetic and electric vectors are not distinguished.

We found that the 5-nm-gap transmission has the same incident angle dependence as the magnetic field intensity at the gold-air interface given by the Fresnel equations (Fig. 2(a), red solid line). This indicates that the transmission decrease at large incident angles is due to the magnetic field decrease on the gold surface. Because the permittivity of gold is finite, the incident magnetic field and the reflected magnetic field destructively interfere to yield the decreased total magnetic field at large incident angles (Supplementary Fig. S1). Given that the tangential magnetic field on a perfect electrical conductor (PEC) film is always twice the incident tangential magnetic field, comparison of the gold and PEC gaps shows this point clearly. Figure 2(b) displays FEM simulation results comparing the transmissions through the gold and PEC gaps of the same 5-nm width. The simulated gold gap transmission decreases at large angles, being consistent with the experiment and the Fresnel equations (Fig. 2(b), red circles). On the other hand, the PEC gap transmission is constant over all incident angles, like the incident magnetic field. In other words, the limiting factor in nanogap optical magnetism is the finite permittivity of a real metal, not the nanogap structure of high aspect ratio beyond the assumption of Babinet’s principle. It should be noted that the metal film should be thicker than the skin depth to block the direct transmission through the metal film. If the film is thinner than the skin depth, the nanogap interacts also with the directly transmitted field^20,21^ and the nanogap optical magnetism disappears (Supplementary Fig. S2).

**Figure 3:**
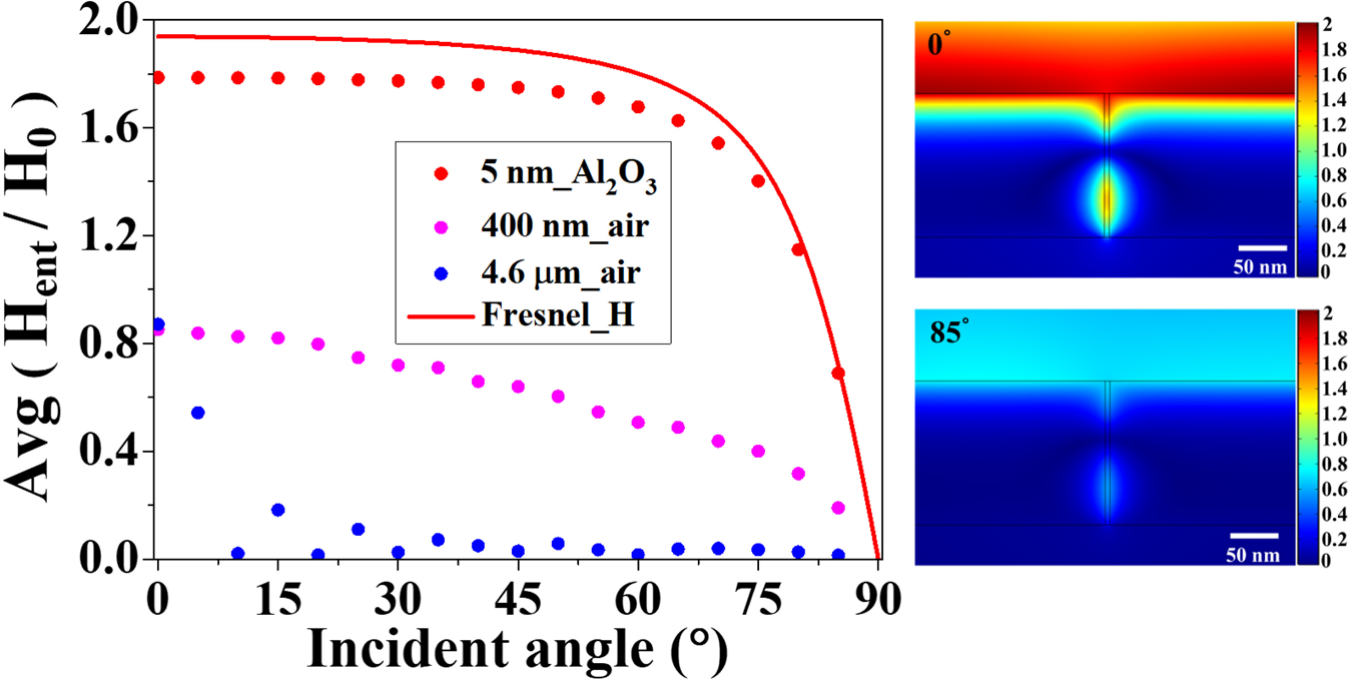
Magnetic field amplitude at the gap entrance versus the incident angle for the 5-nm (red circles), 400-nm (magenta circles) and 4.6-μm (blue circles) gaps. Magnetic field given by the Fresnel equations is shown as the red solid line for comparison. Magnetic field distributions in the vicinity of the 5-nm gap for 0° and 85° incident angles are shown at the right panels. The two cases are the same except for the amplitudes.

For better understanding, we analyzed the relation between the in-gap magnetic field and the magnetic field on the metal surface using FEM simulation. Figure 3 displays the magnetic field amplitudes at the gap entrances with varying incident angle for three different widths of 5 nm, 400 nm, and 4.6 μm. Magnetic field formed on the 5-nm gap keeps up with the Fresnel equations^22^ (Fig. 3, red circles and solid line) in agreement with the experimental results. However, the 400-nm and 4.6-μm gaps, which are almost half and six times the wavelength respectively, have the magnetic field amplitudes irrelevant with the Fresnel equations (Fig. 3, magenta and blue circles), showing that the effect of the metal sides on the in-gap field formation is diminished due to the reduced aspect ratios. Therefore, the dependence of the nanogap transmission on the magnetic field can be interpreted as follows. In the transmission through a subwavelength gap, gap plasmon mediating the transmission is excited by the accumulated charge on the metal sides delivered by the surface current^10,23–25^. Because the surface current is proportionally induced by the tangential magnetic field on the metal surface, the gap transmission has the same incident angle dependence as the tangential magnetic field on the metal surface. Magnetic field distributions of the 5-nm gap for 0° and 85° incident angles presented in the right panels of Fig. 3 further support this interpretation. The two cases show the propagation of the same gap plasmon mode, and thus the transmission is only affected by the amplitude of the tangential magnetic field near the gap.

**Figure 4:**
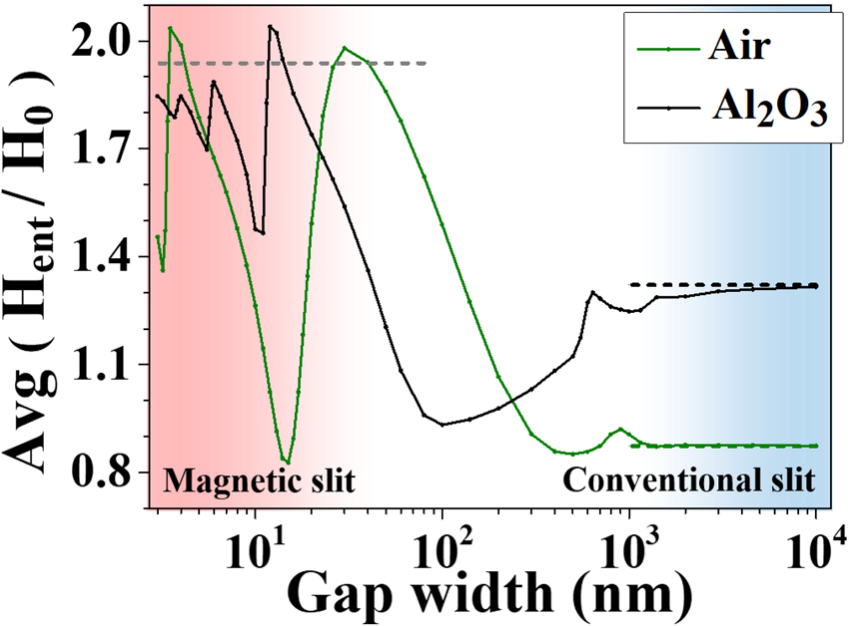
Magnetic field amplitude at the gap entrance versus the gap width with normal incidence for gap dielectrics of Al_2_O_3_ (black solid line) and air (green solid line). For widths below 100 nm, the gap field amplitude oscillates around the value given by the Fresnel equations (gray dashed line). Whereas, the field amplitude in conventional macroscopic slits converges to the value of the dielectric thin film (black and green dashed lines), regardless of the side metal faces.

The range of gap width where the transmission is determined by the incident magnetic field is important in realizing optical magnetism with a nanogap. The width range can be identified based on the above discussion: the range where the gap plasmon dominantly mediates the transmission. We investigated the gap width dependence of the magnetic field amplitude on the gap entrance for normal incidence by FEM simulation. As shown in Fig. 4, when the gap width is under ~100 nm, magnetic field amplitude at the gap entrance oscillates around the amplitude given by the Fresnel equations (Fig. 4, gray dotted line). The oscillation arising under 100-nm width is due to Fabry-Perot interference of the gap plasmon^10,26,27^. Only the fundamental waveguide mode, which is the gap plasmon in our case, excited if the gap width is sufficiently narrow^28^ and the propagation constant of the gap plasmon increases with decreasing the gap width^29^ (Supplementary Fig. S3(a)). Therefore, the emergence of the Fabry-Perot oscillation confirms that the gap plasmon dominantly mediates the transmission, indicating that optical magnetism can be realized up to around 100-nm width for a 150-nm thickness. On the other hand, when the gap width becomes larger, higher order waveguide modes are excited (Supplementary Fig. S3(b)). Because the higher order excitations are not simply proportional to the surface current, a slit wider than ~100 nm cannot exhibit optical magnetism. In a conventional macroscopic slit, currents and charges on the metal sides have only a negligible effect on the in-gap field, making the Kirchhoff approximation valid, and the magnetic field amplitude on the gap converges to that of the dielectric film (Fig. 4, black and green dashed lines).

## Conclusion

We investigated the magnetic nature of light transmission through a 5-nm gap that has an aspect ratio up to 30 by performing the oblique incidence experiment at 785-nm wavelength. The fact that the in-gap fields are determined by the accumulated charge on the metal sides makes the transmission depend on the magnetic field on the metal surface given by the Fresnel equations. In contrast, transmission through a conventional macroscopic slit does not show a specific dependence on the magnetic field and agrees well with the Kirchhoff diffraction theory and the approximation. From the FEM simulation, the range of gap width where optical magnetism takes place is identified to be up to 100 nm for a 150-nm-thick gold film. These results suggest that high aspect ratio nanogap structures provide new possibilities for combining optical magnetism and the enormous in-gap field enhancement to lead novel optical phenomena, for example, magnetic-field-driven optical nonlinearities.

## Methods

### Fabrication

A 150-nm-thick gold film (1st layer) covering half a quartz substrate (Fig. 1(a), x < 0) was deposited by photolithography. The 1st layer then was coated with aluminum oxide (Al_2_O_3_) using atomic layer deposition (ALD). The thickness of the Al_2_O_3_ layer, which specifies the gap width, was controlled by the number of ALD cycles and verified by scanning electron microscopy (Fig. 1(b), inset). After subsequent 150-nm gold deposition (2nd layer), the fabrication was completed by pilling off the 2nd layer which deposited on the top of the 1st layer with scotch tape.

### Simulation

A commercial software package, COMSOL Multiphysics 5.2, was used to conduct the simulations. Refractive indexes for the quartz substrate and the Al_2_O_3_ were set to be 1.45 and 1.7, respectively. The dielectric function for gold was taken from P. B. Johnson and R. W. Christy^30^. The periodicity of the gap was set to be 200 μm which is much longer than the wavelength to see the single slit characteristics. The maximum mesh size in the nanogap was width/15 ensuring the simulation accuracy. The transmission intensity at the exit side was obtained by integrating the z-component of the Poynting vector.

### Kirchhoff’s diffraction theory and approximation

Kirchhoff’s approach^6^ uses Green’s theorem,

1 $$\psi (\vec{x})=\oint [\psi (\vec{x}^{\prime} )\vec{n}^{\prime} \cdot \vec{\nabla }^{\prime} G(\vec{x},\vec{x}^{\prime} )-G(\vec{x},\vec{x}^{\prime} )\vec{n}\cdot \vec{\nabla }^{\prime} \psi (\vec{x}^{\prime} )]da^{\prime} .$$

Because our structure is an infinite slit, Eq. (1) is reduced to a 2-dimensional integration on the incident plane. The Kirchhoff diffraction integral is obtained from Eq. (1) by taking the 2-dimensional Green function as $G(\vec{x},\vec{x}^{\prime} )=\frac{i}{4}{H}_{0}^{(1)}(kr)$. ${H}_{0}^{(1)}(kr)$ is the zeroth-order Hankel function of the first kind where *k* is the wave number and $r=|\vec{x}-\vec{x}^{\prime} |$. In the Kirchhoff approximation, $\psi (\vec{x}^{\prime} )$ is given as the incident field, or ${e}^{i{k}_{x}x^{\prime} }$, at the gap region and vanishes elsewhere. Then the integral reads,

2 $$\psi (\vec{x})=\frac{i}{2}{e}^{-\frac{i\pi }{4}}\sqrt{\frac{k}{2\pi }}[{\int }_{-\,w/2}^{+\,w/2}\frac{{e}^{ikr}}{r}{e}^{i{k}_{x}x^{\prime} }({e}^{-\frac{i\pi }{2}}\,\cos \,{\theta }_{t}-i\,\cos \,{\theta }_{i})dx^{\prime} ]$$

where *w* is the gap width, *θ*_*i*_ is the incident angle, *θ*_*t*_ is the transmitted angle, and ${k}_{x}=k\,\sin \,{\theta }_{i}$. By integrating ${|\psi (\vec{x})|}^{2}$ about the *θ*_*t*_ from −arcsin (*NA*) to +arcsin (*NA*), we can obtain the collected power.

### Fresnel equations

The magnetic field amplitude at the air-metal interface is given as

3 $$\frac{H^{\prime} }{{H}_{0}}=\frac{2nn^{\prime} \cos \,{\theta }_{i}}{{n^{\prime} }^{2}\,\cos \,{\theta }_{i}+n\sqrt{{n^{\prime} }^{2}-{n}^{2}{\sin }^{2}{\theta }_{i}}}$$

where the refractive index of air is *n* and that of the metal film is *n*′. We adopted the same sign convention with the Jackson^6^.

## Electronic supplementary material

Supplementary information
