# Supplementary material for: Magnetic Nature of Light Transmission through a 5-nm Gap
Source: Sci Rep. 2018 Feb 9;8:2751. doi: 10.1038/s41598-018-21037-1 (PMC5807359; doi:10.1038/s41598-018-21037-1)
Supplement: Supplementary file 1 — Supplementary information [file 41598_2018_21037_MOESM1_ESM.pdf]

Supplementary Information

Magnetic Nature of Light Transmission through a 5-nm Gap

Hyosim Yang[1], Dai-Sik Kim[1], Richard H. Joon-Yeon Kim[1,2], Jae Sung Ahn[3], Taehee Kang[1], Jeeyoon Jeong[1], and Dukhyung Lee[*,1]

[1]Department of Physics and Astronomy and Center for Atom Scale Electromagnetism, Seoul National University, Seoul 08826, Republic of Korea

[2]Ames Laboratory, U.S. Department of Energy and Department of Physics and Astronomy, Iowa State University, Ames, Iowa 50011, USA

[3]Bio-medical Photonics Research Center, Korea Photonics Technology Institute, 9 Cheomdan venture-ro 108beon-gil, Gwangju 61007, Republic of Korea

E-mail: hyung0624@snu.ac.kr

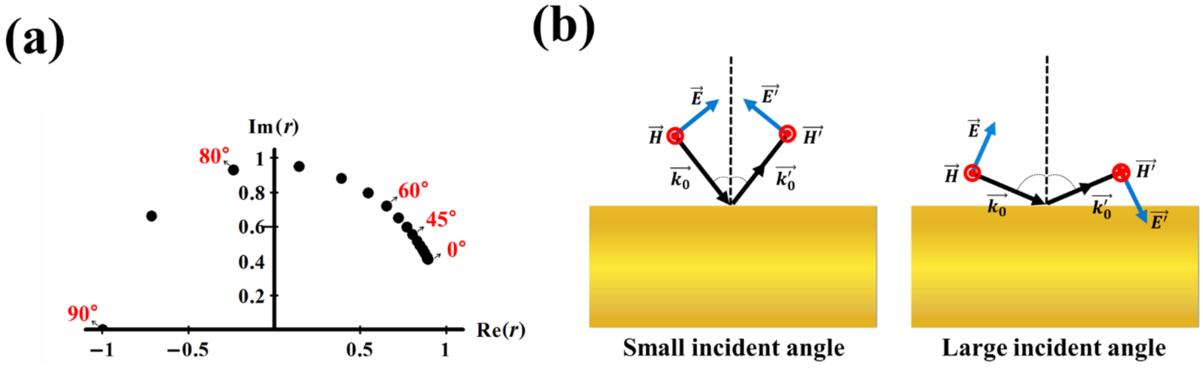

Figure S1. (a) The Fresnel reflection coefficient r for p-polarization plotted in the complex plane for various incident angles. **(b)** Schematic illustrations of the incident and reflected fields at small (left) and large (right) incident angles.

Figure S1(a) indicates that the phase of the Fresnel reflection coefficient r changes with increasing the incident angle. At small incident angles, the reflection coefficient is near 1, which corresponds to constructive interference between the incident magnetic field and the reflected magnetic field. However, from around 60°, the phase starts to change rapidly and, at an incident angle of 90°, the reflection coefficient reaches -1, which corresponds to destructive interference between the incident and reflected magnetic fields. Thus, the magnetic field amplitude on the metal surface decreases in large incident angle as shown in Figs. 2 and 3.

The phase change to the opposite direction can be interpreted in terms of the surface impedance boundary condition (SIBC). The SIBC is given as follows[1]:

$$\vec{E_t} = Z\vec{H_t} \times \vec{n}$$

where Z $(= \sqrt{\mu/\varepsilon} = (1/\tilde{n})Z_0)$ is the surface impedance of the metal $\vec{n}$ is the unit vector of inward normal direction, and $\vec{E_t}$ and $\vec{H_t}$ are the total tangential electric and magnetic fields, respectively. The 'total field' means the superposition of the incident and reflected fields. The SIBC is valid under the conditions that (i) the absolute value of the complex refraction index of the metal $\tilde{n}$ is large compared to unity, and (ii) the penetration depth is small compared with the radii of curvature of the metal surface. Satisfying these conditions, the structure under this study can be treated by the SIBC.

The SIBC indicates that E_t must be $(1/\tilde{n})$ times smaller than Z_0H_t, while $E_0 = Z_0H_0$ in the free space. Therefore, at a small incident angle, the reflection occurs to interfere destructively with the incident tangential electric field and constructively with the incident tangential magnetic field (Fig. S1(b), left). However, if the incident angle is large, the incident tangential electric field $E_0\cos\theta$ becomes minute already. In order to keep the proportional relation of the SIBC, the reflection at a large incident angle occurs to interfere constructively with the incident tangential electric field and destructively with the incident tangential magnetic field (Fig. S1(b), right).

(a)

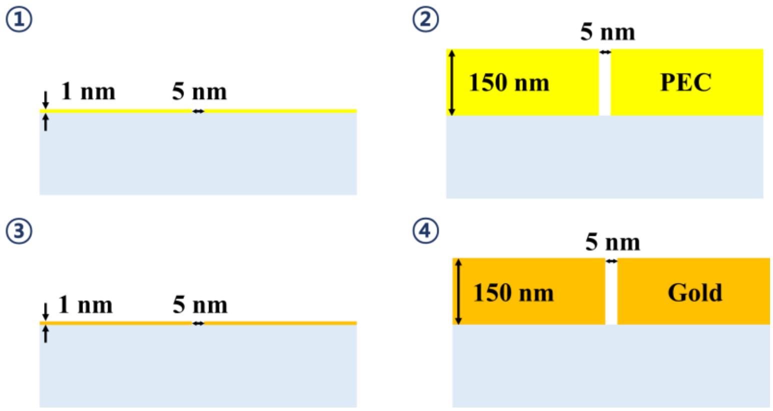

(b)

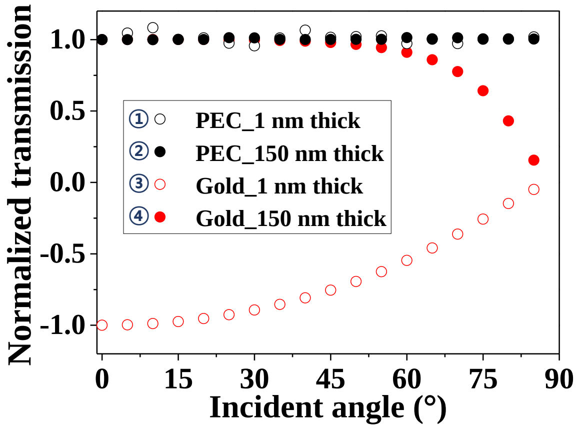

Figure S2. (a) Schematics of the four cases compared to study the effects of the metal permittivity and the film thickness: ① a 5-nm gap on a 1-nm-thick PEC film. ② a 5-nm gap on a 150-nm-thick PEC film. ③ a 5-nm gap on a 1-nm-thick gold film. ④ a 5-nm gap on a 150-nm-thick gold film. The substrate is quartz and the gap dielectric is Al_2O_3. **(b)** Normalized transmissions of the above cases with varying incident angle obtained by FEM simulations.

We examined how the two assumptions of Babinet's principle that the film should be perfectly conducting and infinitely thin determines the optical magnetism of a nanogap. We performed FEM simulations on the above four cases and integrated the poynting vectors at the gap exits. The transmissions keep the almost constant values in both the PEC cases because magnetic field on a PEC film is always twice the incident magnetic field regardless of the film thickness. On the other hand, the transmission through the 5-nm gap on a 150-nm-thick gold film is decreased from 60° due to the finite permittivity as explained in Fig. S1. In case of the 5-nm gap on a 1-nm-thick gold, the transmission is negative due to the destructive interference between the field on the gap and the field directly transmitted through the metal film of the sub-skin depth[2,3]. Therefore, in case of a real metal film, the film thickness should be larger than the skin depth to realize the nanogap optical magnetism.

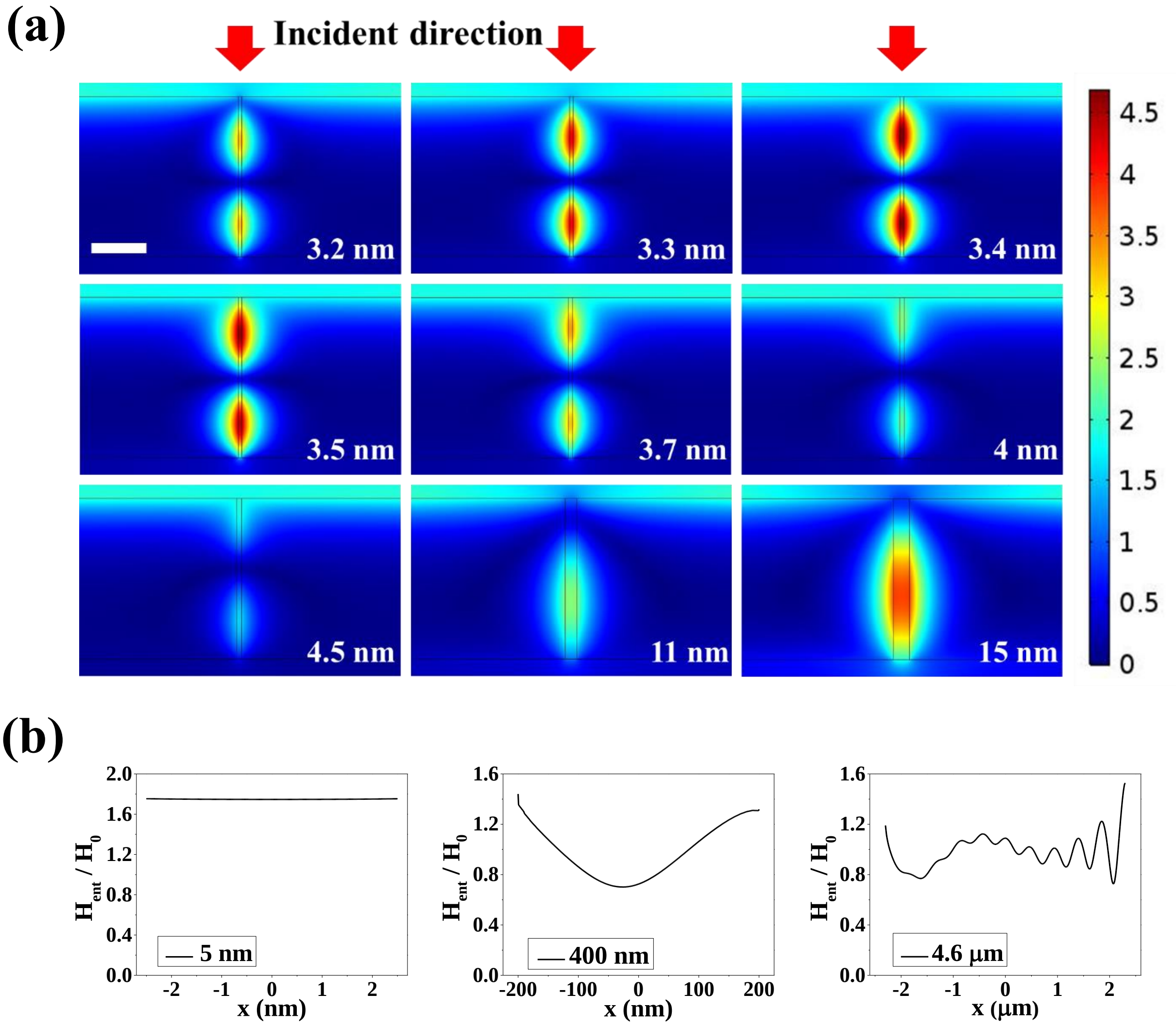

Figure S3. (a) Magnetic field distributions showing Fabry-Perot resonances in the air nanogaps depending on the gap width. The color scale is normalized to the incident amplitude. The white scale bar in the top left panel (3.2 nm gap) represents 50 nm. **(b)** Magnetic field distributions along the width direction at the gap entrances at an incident angle of 45° for gap widths of 5 nm (left), 400 nm (middle), and 4.6 μm (right).

Figure S3(a) presents the magnetic field distributions showing Fabre-Perot resonances depending on the gap width. The effective wavelength of the gap plasmon gets shorter as the gap widths decrease. The spatial shift of the nodes and the anti-nodes is responsible for the amplitude oscillation in Fig. 4. Figure S3(b) shows the magnetic field amplitudes at the gap entrances for the gap widths of 5 nm, 400 nm, and 4.6 μm. Only the fundamental mode is excited in the 5-nm gap and the multi-modes are excited in the wider gaps.

References

(1) Landau, L. D., Lifshitz, E. M., *Electrodynamics of Continuous Media.* (Addison-Wesley, 1960).

(2) Braun, J., Gompf, B., Kobiela, G. & Dressel, M. How Holes Can Obscure the View: Suppressed Transmission through an Ultrathin Metal Film by a Subwavelength Hole Array. *Phys. Rev. Lett.* **103**, 203901 (2009).

(3) Spevak, I. S., Nikitin, A. Y., Bezuglyi, E. V., Levchenko, A. & Kats, A. V. Resonantly suppressed transmission and anomalously enhanced light absorption in periodically modulated ultrathin metal films. *Phys. Rev. B* **79**, 161406 (2009).
